# CRISPR/Cas9-mediated PD-1 disruption enhances anti-tumor efficacy of human chimeric antigen receptor T cells

**DOI:** 10.1038/s41598-017-00462-8

**Published:** 2017-04-07

**Authors:** Levi J. Rupp, Kathrin Schumann, Kole T. Roybal, Rachel E. Gate, Chun J. Ye, Wendell A. Lim, Alexander Marson

**Affiliations:** 1 Department of Cellular & Molecular Pharmacology, San Francisco, CA USA; 2 Center for Systems and Synthetic Biology, University of California San Francisco, San Francisco, 94158 CA USA; 3 Helen Diller Family Comprehensive Cancer Center, University of California San Francisco, San Francisco, CA 94158 USA; 4 Howard Hughes Medical Institute, University of California San Francisco, San Francisco, 94158 CA USA; 5 Diabetes Center, University of California San Francisco, San Francisco, CA 94143 USA; 6 Department of Microbiology and Immunology, University of California San Francisco, San Francisco, CA 94143 USA; 7 Department of Epidemiology and Biostatistics, Department of Bioengineering and Therapeutic Sciences, Institute for Human Genetics, University of California, San Francisco, CA 94143 USA; 8 Biological and Medical Informatics Graduate Program, University of California, San Francisco, CA 94158 USA; 9 Division of Infectious Diseases and Rheumatology, Department of Medicine, University of California San Francisco, San Francisco, CA 94143 USA; 10 Innovative Genomics Institute, University of California Berkeley, Berkeley, CA 94720 USA

## Abstract

Immunotherapies with chimeric antigen receptor (CAR) T cells and checkpoint inhibitors (including antibodies that antagonize programmed cell death protein 1 [PD-1]) have both opened new avenues for cancer treatment, but the clinical potential of combined disruption of inhibitory checkpoints and CAR T cell therapy remains incompletely explored. Here we show that programmed death ligand 1 (PD-L1) expression on tumor cells can render human CAR T cells (anti-CD19 4-1BBζ) hypo-functional, resulting in impaired tumor clearance in a sub-cutaneous xenograft model. To overcome this suppressed anti-tumor response, we developed a protocol for combined Cas9 ribonucleoprotein (Cas9 RNP)-mediated gene editing and lentiviral transduction to generate PD-1 deficient anti-CD19 CAR T cells. *Pdcd1* (PD-1) disruption augmented CAR T cell mediated killing of tumor cells *in vitro* and enhanced clearance of PD-L1+ tumor xenografts *in vivo*. This study demonstrates improved therapeutic efficacy of Cas9-edited CAR T cells and highlights the potential of precision genome engineering to enhance next-generation cell therapies.

## Introduction

Chimeric antigen receptor (CAR) T cells have shown promising clinical results against multiple classes of B cell leukemias and lymphomas, but challenges still remain in targeting certain liquid and solid tumors^1^. The recent advent of readily programmable methods for CRISPR/Cas9-based gene editing of primary human T cells^2–4^ raises the prospect of enhanced CAR T cell therapy via gene modification and/or disruption. In this study we first explored whether expression of the inhibitory receptor PD-L1 on tumor cells could suppress highly potent anti-CD19 CAR T cells. We subsequently developed a protocol for combined Cas9-based gene disruption and lentiviral transduction to generate gene modified human CAR T cells, and then tested whether Cas9-mediated disruption of PD-1 in CAR T cells improved anti-tumor efficacy *in vitro* and *in vivo* using a xenograft tumor model.

Chimeric antigen receptor (CAR) T cells are genetically engineered lymphocytes that express a synthetic receptor comprised of an extracellular antigen recognition domain (typically a single chain variable fragment [scFv] recognizing a tumor antigen) fused to intra-cellular domains that recapitulate signaling events downstream of endogenous T cell receptor (TCR) activation^5^. So-called “second generation” CARs encode both the CD3ζ chain and motifs from costimulatory proteins such as CD28 or 4-1BB (CD137) that promote T cell proliferation and survival. While CAR T cells have demonstrated potent anti-tumor capacity in leukemia and lymphoma, efficacy in some liquid tumors and many solid tumors has been lacking^1^. One mechanism by which both liquid and solid tumors can inhibit T cell function and efficacy is via an immunosuppressive tumor microenvironment and expression of inhibitory ligands such as PD-L1 on both tumor cells and surrounding tissues (e.g. stroma or tumor vasculature)^6–8^.

The PD-1/PD-L1 axis is a critical regulator of T cell fate and function. PD-1 is transiently up-regulated on T cells following activation but has also been identified as a marker of T cell exhaustion, a hypo-functional cell state found in chronic viral infections and amongst tumor infiltrating lymphocytes in patients with advanced malignancy^9, 10^. Notably, expression of the PD-1 ligands PD-L1 and PD-L2 is correlated with poor prognosis in multiple tumors^11, 12^ and anti-PD-1/anti-PD-L1 blocking antibodies have been shown to induce potent anti-tumor immune responses in patients with diverse malignancies^13, 14^, demonstrating the critical role of the PD-1/PD-L1 axis in anti-tumor immunity.

We wanted to test whether ablating *Pdcd1* specifically in CAR T cells would allow for generation of tumor-specific cells with enhanced anti-tumor functionality. Despite the clear role of PD-1/PD-L1 in regulating endogenous anti-tumor responses, the impact of inhibitory receptors on CAR T cell function remains largely unexplored. John *et al.* demonstrated that antibody-mediated PD-1 blockade enhanced CAR T cell function in a syngeneic mouse model^15^, but at least part of this effect was mediated through inhibition of myeloid-derived suppressor cells (MDSCs) that express PD-1, rather than direct impact on CAR T cells. Fedorov *et al.* have shown that recruitment of the PD-1 intracellular domain through synthetic inhibitory CARs (iCARs) can suppress activity of CAR T cells^16^, suggesting that PD-1 ligation might inhibit CAR function. Notably, both this study and John *et al.* employed CD28ζ CARs rather than 4-1BBζ CARs. Thus, the cell autonomous effect of endogenous PD-1/PD-L1 ligation on human 4-1BBζ CAR T cells *in vivo* remains to be determined. Moreover, while combination therapy with PD-1 blockade and CAR T cells could enhance CAR T cell function, systemic PD-1 blockade is associated with toxicities due to enhanced activation of autoreactive T cells^13^. Ablation of *Pdcd1* specifically in CAR T cells might therefore provide a safer way to overcome tumor immunosuppression, particularly when combined with TCR disruption to prevent activation of autoreactive T cells^17^.

In this study we tested whether Cas9 RNP mediated disruption of the endogenous *Pdcd1* locus in primary human CAR T cells enhances anti-tumor efficacy. We found that PD-L1 expression on tumor cells impaired CAR T cell mediated killing *in vitro* and tumor clearance *in vivo* in a xenograft model. These defects could be mitigated by Cas9-mediated *Pdcd1* disruption within the CAR T cells. CRISPR-mediated gene editing combined with lentiviral transduction of CAR T cells was highly efficient, and raises the prospect of further complex engineering of cell therapy products to enhance safety and anti-tumor efficacy.

## Results

### PD-L1 expression on tumor cells impairs CAR T cell function *in vitro*

**Figure 1:**
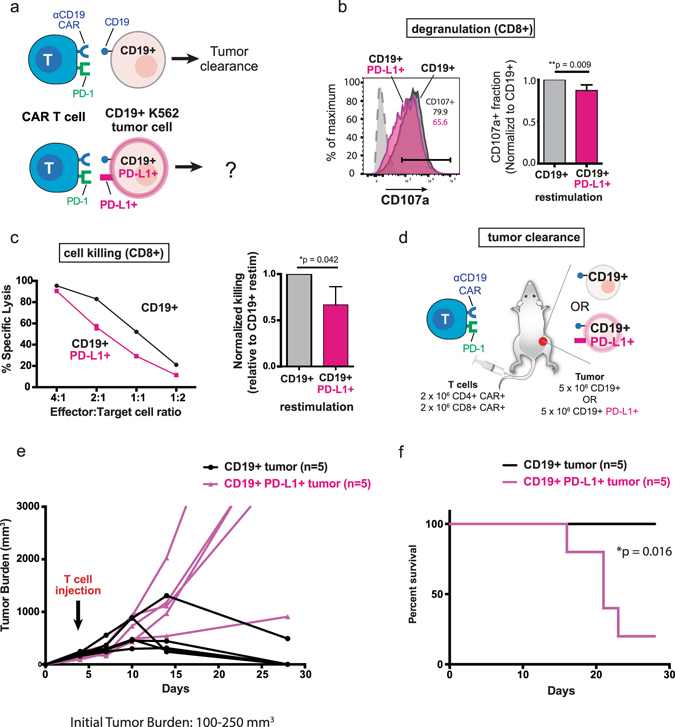
PD-L1 expression in human K562 myelogenous leukemia cells inhibits anti-CD19 CAR T cell function *in vitro* and tumor clearance *in vivo*. (**a**) Schematic representation of CAR T cell interaction with either CD19+ or CD19+ PD-L1 K562 tumor cells. (**b**) CD8+ anti-CD19 CAR T cells exhibit reduced degranulation (CD107a staining) upon re-stimulation with CD19+ PD-L1+ K562 cells. Representative CD107a staining is shown, gated on CD8+ CAR+ T cells. Normalized CD107a staining shows ~15% reduction in degranulation upon co-culture with CD19+ PD-L1+ targets over three independent experiments (**p = 0.009, Student’s t-test). (**c**) CD19+ PD-L1+ K562 cells are resistant to anti-CD19 CAR-mediated lysis in an *in vitro* killing assay. Left panel: a complete effector:target ratio titration is shown for a representative experiment. Mean ± S.D. for triplicate wells in a single experiment are plotted. Right panel (bar chart): CD19+ PD-L1+ K562 cells induce ~40% reduction in specific lysis relative to CD19+ K562 cells at effector:target ratio of 2:1 (*p = 0.042, Student’s t-test). The experiment was performed three times; error bars are S.D. (**d**) Experimental design for subcutaneous xenograft model. (**e**) CD19+ PD-L1+ subcutaneous xenografts impair anti-CD19 CAR mediated tumor clearance. NOD-*scid*-IL-2Rγ^−/−^ (NSG) mice were injected with 5 × 10^6^ CD19+ or CD19+ PD-L1+ K562 cells subcutaneously. Mice with established tumors (100–250 mm^3^) were injected intravenously with 2 × 10^6^ CD4+ and 2 × 10^6^ CD8+ anti-CD19 CAR T cells and tumor burden measured longitudinally by caliper. Shown are tumor burdens for individual mice (n = 5 per tumor type). (**f**) Kaplan-Meier curve for experiment described in Fig. 1E. A statistically significant difference in survival was observed between animals bearing CD19+ vs. CD19+ PD-L1+ tumors (*p = 0.016, Gehan-Breslow-Wilcoxon test).

While the PD-1/PD-L1 pathway has been shown to modulate endogenous TCR signaling and downstream T cell functions^10, 18^, we first wished to determine whether chimeric antigen receptor (CAR) T cells were sensitive to PD-1 pathway ligation. To this end we generated a PD-L1 expressing tumor cell line by transducing CD19+ K562 myelogenous leukemia cells^19^ with a lentiviral vector expressing human PD-L1, to generate CD19+ PD-L1+ K562 cells (Fig. 1a and Supplementary Fig. 1a). Critically, K562 cells also lack detectable surface expression of MHC I (Supplementary Fig. 1b), thereby precluding any confounding effects of TCR-mediated signaling in CD8+ CAR T cells. We utilized a second generation anti-CD19 4-1BBζ CAR (aCD19 CAR) for these studies because it has displayed potent anti-tumor activity in clinical studies^20^ and we hypothesized that it would present a stringent challenge for PD-L1 mediated suppression of CAR T cell function.

CAR T cells were generated by activating primary human CD8+ cytotoxic T cells with anti-CD3/anti-CD28 beads and transducing activated cells with a lentiviral vector encoding the anti-CD19 CAR. CD8+ T cells expressing the anti-CD19 CAR were allowed to rest and then re-stimulated with either CD19+ or CD19+ PD-L1+ K562 cells and assayed for canonical effector functions. CD8+ CAR T cells re-stimulated with CD19+ PD-L1+ targets exhibited an ~15% reduction in degranulation (CD107a staining) in a 5-hour co-culture assay (Fig. 1b, **p = 0.009). Consistent with this result, CD19+ PD-L1+ target cells were 10–40% less susceptible to anti-CD19 CAR T cell mediated lysis in an overnight killing assay (Fig. 1c, *p = 0.042 at effector:target ratio of 2:1). We observed that the magnitude of PD-L1-mediated inhibition of lysis was variable across T cell donors and different effector:target ratios, but CD19+ PD-L1+ cells were consistently resistant to CAR-mediated lysis across multiple donors and effector:target ratios (Fig. 1c). Combined, these results suggest that PD-L1 expression on tumor cells can directly impair the function and lytic capacity of primary human CD8+ T cells expressing the anti-CD19 4-1BBζ CAR.

### anti-CD19 CAR T cells exhibit impaired clearance of subcutaneous PD-L1+ tumor xenografts

We next aimed to determine whether PD-L1-mediated impairment of CAR T cell function impacted tumor clearance *in vivo*. NOD-*scid*-IL-2Rγ^−/−^ (NSG) mice were injected subcutaneously with 5 × 10^6^ CD19+ or CD19+ PD-L1+ K562 cells and tumors were established at ~100–250 mm^3^ prior to adoptive transfer of 2 × 10^6^ CD4+ and 2 × 10^6^ CD8+ anti-CD19 CAR T cells (Fig. 1a). We employed both CD4+ and CD8+ CAR T cells for *in vivo* experiments to mimic current clinical protocols for CAR therapy.

At the indicated dose and tumor burden we observed clearance of CD19+ tumors in 80% of mice by day 28 of the experiment. In contrast, 80% of animals bearing CD19+ PD-L1+ tumors had succumbed to disease and had to be euthanized (Fig. 1e and f, *p = 0.016, Gehan-Breslow-Wilcoxon test). In other experiments conducted with different T cell doses and initial tumor burdens (Supplementary Fig. 2a,b) we observed a similar trend, in that a proportion of mice bearing CD19+ PD-L1+ tumors either failed to clear tumors and succumbed to disease, or had delayed clearance/larger tumor burdens relative to animals with control CD19+ tumors. Combined, these results indicate that CD19+ PD-L1+ subcutaneous tumor xenografts can inhibit anti-CD19 CAR mediated tumor clearance.

Recent reports have indicated that PD-1/PD-L1 signaling in tumor cells can alter the metabolism and intrinsic growth rate of tumor cells^21, 22^. Despite PD-1 expression on a fraction of the parental CD19+ K562 cell line (Supplementary Fig. 3a), there was no difference in growth kinetics of CD19+ and CD19+ PD-L1+ cells either *in vitro* (Supplementary Fig. 3b) or *in vivo* (Supplementary Fig. 3c), suggesting that ectopic PD-L1 expression did not significantly alter intrinsic tumor growth in this model^22^. These experiments demonstrate that tumor-specific PD-L1 expression can inhibit anti-CD19 CAR-mediated clearance of established subcutaneous tumor xenografts. This xenotransplant system also serves as a useful *in vivo* model to study the mechanisms by which PD-1/PD-L1 signaling can inhibit human CAR T cell function.

### Efficient generation of PD-1 deficient human CAR T cells with Cas9 RNPs

**Figure 2:**
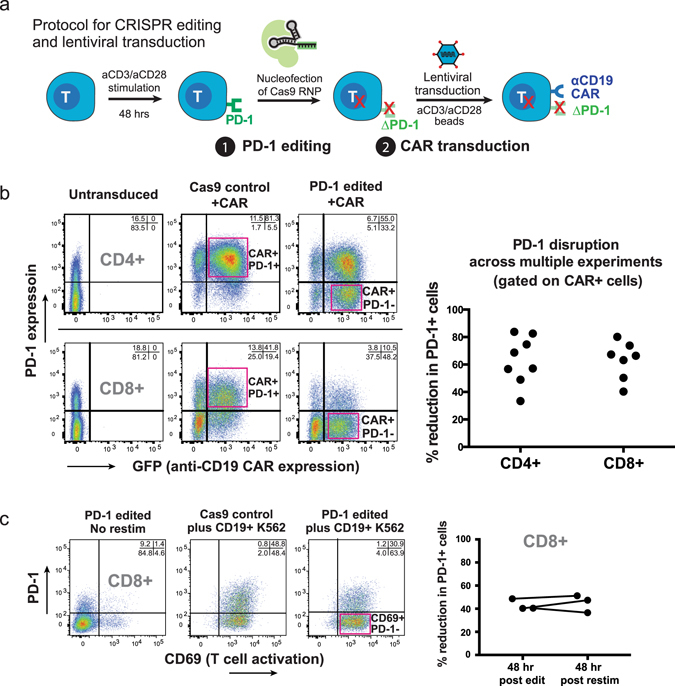
*Pdcd1* can be efficiently disrupted in CAR T cells using Cas9 ribonucleoproteins (Cas9 RNPs). (**a**) Schematic of protocol for combined CRISPR gene editing and lentiviral transduction of human primary T cells. (**b**) Efficient PD-1 deletion and CAR transduction in primary human T cells. PD-1 surface staining and CAR transduction were assessed 48 hours post editing. A >50% reduction in PD-1+ cells was routinely observed, with CAR transduction >70%. Right panel, individual dots represent independent editing experiments. (**c**) PD-1 edited CAR T cells are stable in culture. Resting PD-1 edited CD8+ anti-CD19 CAR T cells were re-stimulated with CD19+ K562 cells. Activation was measured by CD69 induction and percent reduction in PD-1+ cells measured by flow cytometry based on surface PD-1 expression; percent reduction of PD-1+ cells was similar to that observed 48 hours after editing (rightmost panel, pairwise plot).

We hypothesized that genetic disruption of *Pdcd1* in CAR T cells might be sufficient to rescue the observed impairment of anti-tumor activity against PD-L1+ cancer cells. To this end we devised a protocol for combined Cas9 RNP gene editing and lentiviral transduction of primary human T cells. We have previously shown that electroporation of Cas9 ribonucleoproteins (RNPs) mediates efficient gene editing in primary human T cells^3^. We modified the original Cas9 RNP protocol to permit combined gene editing and lentiviral transduction (Fig. 2a). Briefly, primary human T cells were stimulated for 48 hours with plate-bound anti-CD3 and soluble anti-CD28, nucleofected with Cas9 alone (Cas9 control) or Cas9 pre-loaded with chemically synthesized crRNA:tracrRNA duplex targeting *Pdcd1* exon 1^3^, and subsequently transduced with lentiviral vector encoding the anti-CD19 CAR. Cells were then cultured with anti-CD3/anti-CD28 beads for an additional 4–6 days to enhance expansion.

PD-1 ablation efficiency and lentiviral CAR transduction were measured by flow cytometry 2–4 days following nucleofection. We routinely observed a >50% reduction of CAR+ PD-1+ cells 48 hours post-editing, and PD-1 could be successfully ablated in both CD4+ and CD8+ cells (Fig. 2b). Deep sequencing of Cas9 control and PD-1 edited samples revealed significant levels of insertions/deletions (INDELs) at the target locus specifically in PD-1 edited samples (Supplementary Fig. 4); deletions were the most prominent modification, consistent with previous results^3^.

We did not observe any difference in PD-1 ablation frequency between CAR+ and CAR- T cells, indicating the two processes are independent. To determine whether PD-1 deletion had any detrimental effect on CAR T cell activation, rested bulk PD-1 edited CD8+ CAR T cells were re-stimulated with CD19+ K562s and T cell activation monitored by CD69 and PD-1 expression. PD-1 deletion did not impact CAR-mediated activation, as evidenced by uniform CD69 up-regulation in both edited and control CD8+ CAR T cells following re-stimulation (Fig. 2c). Additionally, the reduction in PD-1+ cells in edited cultures 48 hours post re-stimulation was similar to that observed 48 hours after nucleofection, suggesting PD-1 deficient cells were stable during anti-CD3/anti-CD28 mediated expansion following nucleofection (Fig. 2c).

We also measured expansion of Cas9 control or PD-1 edited CAR T cells following electroporation to determine if PD-1 targeting affected proliferation and/or survival of CAR T cells. In most experiments we noticed no difference in expansion between control and PD-1 targeted cells (Supplementary Fig. 5a), but in some experiments PD-1 targeted cells exhibited reduced expansion (Supplementary Fig. 5b). When analyzed across multiple experiments and donors, however, there was no statistically significant difference in expansion between control and PD-1 targeted CAR T cells at days 9 or 11 post-stimulation (Supplementary Fig. 5c,d; paired t-test). Combined, these results indicate that primary T cells can be subjected to combinatorial high efficiency CRISPR-editing and lentiviral transduction to robustly generate PD-1 deficient CAR T cells.

### PD-1 edited CAR T cells exhibit enhanced anti-tumor efficacy

**Figure 3:**
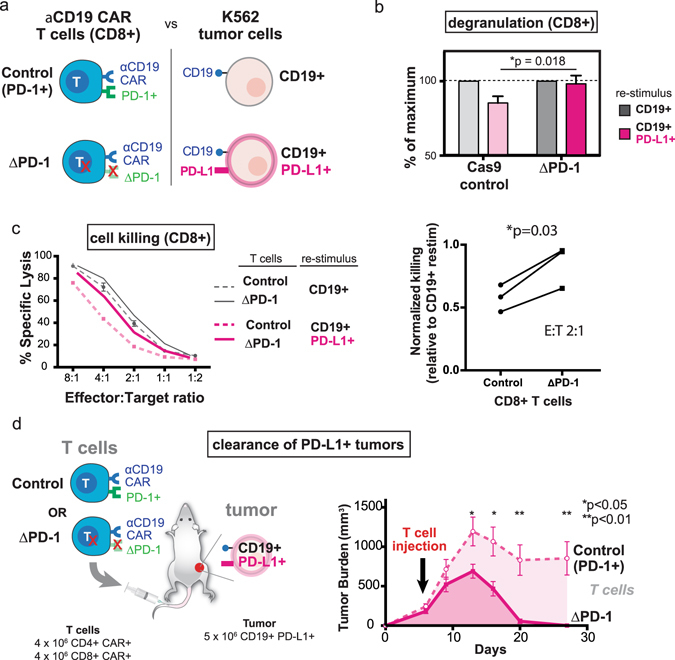
CRISPR-mediated PD-1 editing rescues anti-CD19 CAR T cell function *in vitro* and enhances tumor clearance *in vivo*. (**a**) Diagram of PD-1 edited CAR T cell: K562 interactions (**b**) PD-1 edited CD8+ anti-CD19 CAR T cells (ΔPD-1) exhibit greater degranulation (CD107a staining) upon co-culture with CD19+ PD-L1+ K562 cells as compared to control CD8+ CAR T cells (*p = 0.018, Student’s t-test). (**c**) PD-1 edited CAR T cells are partially resistant to CD19+ PD-L1+ mediated inhibition of cytolysis. Left panel: percent lysis for control and PD-1 edited CD8+ anti-CD19 CAR T cells is shown across a range of effector:target ratios. Error bars are S.D. of triplicate wells in a single experiment. Right panel: normalized killing shows reduced PD-L1 dependent inhibition of killing in PD-1 edited CD8+ CAR T cells at effector:target ratio of 2:1 (*p = 0.03, paired t-test). The experiment was performed three independent times. (**d**) PD-1 deficient anti-CD19 CAR T cells exhibit enhanced anti-tumor efficacy and clear subcutaneous CD19+ PD-L1+ tumor xenografts. NSG mice were injected with 5 × 10^6^ CD19+ PD-L1+ K562 cells subcutaneously. Mice with established tumors (100–250 mm^3^) were injected intravenously with 4 × 10^6^ CD4+ CAR+ and 4 × 10^6^ CD8+ CAR+ control T cells or PD-1 edited cells, and tumor burden measured longitudinally by caliper. Tumor burdens are mean ± SEM for each group (n = 6 mice per group). A statistically significant decrease in tumor burden of mice receiving PD-1 edited CAR T cells was observed at multiple points (*p < 0.05, **p < 0.01, Student’s t-test).

To determine the effect of PD-1 deficiency on CAR T cell function, we first assayed PD-1 edited anti-CD19 CAR T cells *in vitro* (Fig. 3a). These studies were performed on bulk populations that contained both edited and un-edited cells. As shown in Fig. 3b, *Pdcd1* disruption (ΔPD-1) rescued the defect in degranulation (CD107a expression) observed following stimulation of CD8+ CAR T cells with CD19+ PD-L1+ tumor cells, in contrast to control Cas9 nucleofected cells. We also tested the cytolytic capacity of PD-1 edited anti-CD19 CAR T cells against both CD19+ and CD19+ PD-L1+ targets. Consistent with the degranulation results, PD-1 edited CD8+ CAR T cells were more efficient at killing PD-L1+ tumor cells relative to control Cas9 nucleofected cells (Fig. 3c, *p = 0.03 at E:T ratio of 2:1). In sum, these results suggest that *Pdcd1* disruption can rescue functional defects induced in CD8+ anti-CD19 4-1BBζ CAR T cells by PD-L1+ tumor cells.

Finally, we tested whether *Pdcd1* disrupted CAR T cells exhibited enhanced anti-tumor efficacy *in vivo*. NSG mice were injected subcutaneously with CD19+ PD-L1+ K562 cells and tumors were established before injection with either PD-1 edited or Cas9 control (non-edited) anti-CD19 CAR T cells (4 × 10^6^ CD4+ CAR+ and 4 × 10^6^ CD8+ CAR+ cells). Bulk T cell populations were employed in these experiments, with no enrichment for PD-1 edited cells.

Mice with initial tumor burdens of 100–250 mm^3^ displayed the greatest difference in clearance; 100% of animals receiving PD-1 edited CAR T cells were able to clear tumors, whereas only 1 of 6 (~17%) animals treated with control anti-CD19 CAR T cells exhibited clearance at 28 days post tumor implant (Fig. 3d and Supplementary Fig. 6). The overall outcome of disease was dependent upon the number of transferred CAR T cells; when mice with 100–250 mm^3^ CD19+ PD-L1+ tumors were treated with 2 × 10^6^ CD4+ CAR+ and 2 × 10^6^ CD8+ CAR+ cells we did not observe clearance of CD19+ PD-L1+ tumors in mice receiving either control or PD-1 edited cells. Median survival of animals treated with PD-1 edited cells was 35 days vs. 21 days for animals receiving control cells (Supplementary Fig. 7), although this difference was not statistically significant (p = 0.06, Gehan-Breslow-Wolcoxon test). Finally, we observed accelerated tumor clearance by PD-1 edited CAR T cells relative to controls (Supplementary Fig. 8a,b) when animals with smaller initial tumors (50–100 mm^3^) were treated with a larger number of CAR T cells (5 × 10^6^ CD4+ CAR+ and 5 × 10^6^ CD8+ CAR+ cells), although we noted that both un-edited and edited cells were able to clear CD19+ PD-L1+ tumors at this low tumor burden and high T cell dose. Combined, our results indicate that CRISPR-mediated targeted disruption of the *Pdcd1* locus can enhance *in vivo* anti-tumor efficacy of human CAR T cells.

## Discussion

In this study we show that tumor-specific PD-L1 expression can render second generation anti-CD19 4-1BBζ CAR T cells hypo-functional *in vitro* and impair tumor clearance *in vivo* in a subcutaneous tumor xenograft model. We subsequently developed a protocol for combinatorial Cas9 RNP editing and lentiviral transduction and generated *Pdcd1* deficient anti-CD19 CAR T cells. Cas9-mediated *Pdcd1* knockout rescued many of the *in vitro* defects observed upon co-culture of CAR T cells with PD-L1+ tumors and ultimately allowed CAR T cells to clear PD-L1+ tumors *in vivo*. These results demonstrate the inhibitory role of the PD-1/PD-L1 axis on CAR T cell anti-tumor function, and demonstrate proof-of-principle for Cas9-based gene editing to enhance CAR T cell efficacy.

This work raises a number of questions to be addressed in future studies. Studies of T cell receptor (TCR) signaling have shown that PD-1 ligation can dampen Akt pathway signaling, amongst other effects^18, 23^. However, the direct mechanisms by which PD-1/PD-L1 ligation results in impaired CAR T cell function remain to be elucidated. By pinpointing the level at which PD-1 ligation impairs CAR T cell signaling or function, it might be possible to engineer CARs with additional or alternative costimulatory domains that are resistant to PD-L1 mediated immunosuppression. Given that CAR T cells with different costimulatory domains (such as those derived from CD28, 4-1BB, or ICOS) exhibit differential activity, phenotypes, and PD-1 expression^24–26^, it will be critical to determine whether *Pdcd1* deficiency has differential effects based upon the specific CAR employed.

Our *in vitro* studies focused on the effects of *Pdcd1* disruption in CD8+ T cells because the link between the PD-1/PD-L1 axis and CD8+ T cell exhaustion is well established^10^. Our *in vivo* experiments employed *Pdcd1* edited CD4+ and CD8+ T cells, however, as this is standard practice for current CAR T cell therapies being tested clinically. Amarnath *et al*. have demonstrated that co-culture/adoptive transfer of human Th1 polarized CD4+ T cells with PD-L1+ targets can re-program Th1 cells to immunosuppressive Tregs^27^. It is therefore possible that the impaired tumor clearance we observed with PD-L1+ tumors is due in part to generation of immunosuppressive Tregs, and *Pdcd1* deletion in CD4+ T cells abrogates this re-programming and enhances tumor clearance. In the future it will be important to explore the impact of *Pdcd1* disruption in both CD4+ and CD8+ CAR T cells singly and in combination.

Another variable that remains to be addressed is the impact of varying the proportion of *Pdcd1* deficient CAR T cells transferred. This is particularly relevant given recent results in the mouse lymphocytic choriomeningitis virus (LCMV) model of chronic infection where Odorizzi *et al*. demonstrated that genetic deletion of *Pdcd1* in naive CD8+ T cells unexpectedly led to increased exhaustion and impaired CD8 T cell survival and function^28^. This is in contrast to anti-PD-1 antibody treatment, which has been shown to rescue T cell function in the LCMV model^9^. These results suggest it is possible that robust deletion of *Pdcd1* could enhance short-term functions of CAR T cells but ultimately render edited cells more susceptible to exhaustion/impaired function (for example, if the tumor is not rapidly cleared). Such an outcome could manifest as an increased incidence of relapse in animals receiving higher proportions of *Pdcd1* edited cells, but the subcutaneous K562 xenografts employed here uniformly relapse as CD19^neg^ tumors (data not shown), precluding this analysis in our model system. Ultimately, further investigations into the interplay of tumor burden, T cell number, and editing frequency in diverse tumor models are warranted.

Our ectopic PD-L1 expressing tumor model also illustrates the power of engineered human xenografts to study the impact of specific biological pathways in isolation. Further characterization of T cells in the CD19+ vs. CD19+ PD-L1+ model could help unravel the mechanisms by which tumor-specific PD-L1 expression impairs CAR T cell function. Additionally, construction of more complex models with expression of multiple inhibitory receptors could be employed to systematically examine the impact of inhibitory receptor signaling on CAR T cell function.

The ability to robustly and efficiently perform genetic manipulations of primary human T cells opens diverse avenues for future study and improved adoptive immunotherapy. For example, the enhanced anti-tumor functionality of *Pdcd1* edited CAR T cells might allow physicians to administer fewer cells to achieve equivalent therapeutic effects. Given that CAR T cell expansion and engraftment are strongly correlated with therapeutic outcome^1^, reducing the number of cells required for efficacy could be a particular boon for patients with cancers such as lymphoma, which is associated with impaired expansion during the CAR T cell manufacturing protocol^29^. A potential caveat to our approach is that *Pdcd1* deficient CAR T cells could express auto-reactive TCRs, resulting in autoimmune side-effects similar to those observed with systemic PD-1 antibody blockade^13^. However, other groups have reported generation of “universal” allogeneic T cells through deletion of endogenous TCR genes to prevent graft-versus-host disease^17, 30, 31^. Given the ease of multiplexed CRISPR/Cas9 based editing^32^, addition of guide RNAs targeting the endogenous TCR and selective depletion of TCR+ cells should allow generation of highly potent, tumor specific CAR T cells lacking any capacity to target non-CAR antigens.

A prominent side-effect of anti-CD19 CAR T cell therapy in leukemia patients is cytokine release syndrome (CRS) mediated by excessive T cell activation^20^. In this regard, enhancing activity of aCD19 CAR T cells via *Pdcd1* deletion could be undesirable, although protocols for clinical management of CRS are continually evolving^20^. In many other indications, however, CAR T cell activity is insufficient to induce robust tumor clearance, and our work demonstrates proof-of-principle for genetic disruption of inhibitory checkpoints as a means to enhance CAR T cell function. Finally, prior to clinical application of CRISPR/Cas9 based therapeutics it will be vital to interrogate off-target cleavage and genotoxicity of given guide RNAs. Recent developments in identifying genome-wide off-target cleavage events^33^ and engineering Cas9 nucleases for enhanced specificity^34, 35^ should aid in addressing these issues.

Robust Cas9 RNP based editing of T cells should also enable studies of basic human T cell biology; the ease of reprogramming genomic loci and potential for screening^36^ could aid in identification of novel targets that further enhance anti-tumor T cell function. Additionally, knock-in genetic alterations^3^ could be employed to explore gain of function mutations or re-wire endogenous circuits to generate enhanced cellular therapies for diverse diseases. The ability to perform targeted genome engineering of human tumor-specific T cells and interrogate the functional consequences *in vitro* and *in vivo* should accelerate the next generation of immunotherapies for cancer.

## Materials and Methods

### Experimental Design

Unless otherwise specified, all experiments were performed at least three times with at least two independent T cell donors.

### Mouse studies

All animal studies were approved by the University of California, San Francisco Institutional Animal Care and Use Committee, and conducted in accordance with institutional guidelines.

### T cell isolation and culture conditions

Human T cells were obtained from anonymous blood donors (Blood Centers of the Pacific, San Francisco, CA) following written informed consent. All studies were conducted in accordance with University of California, San Francisco guidelines. Purified human CD4+ or CD8+ T cells were isolated via negative selection (EasySep) as described^19^. T cells were cryopreserved in RPMI supplemented with 20% human AB serum and 10% DMSO. Upon thawing, cells were rested overnight in T cell medium supplemented with IL-2 (30 U/mL), prior to activation. T cell medium (TCM): X-Vivo 15 (Lonza), 5% human AB serum (Valley Biomedical), 55 μM β-mercaptoethanol, 10 mM N-acetyl L-cysteine.

### Lentivirus production

Lentiviral vector production was performed as previously described^19^. The anti-CD19 4-1BBζ CAR construct was a gift from Michael Milone at the University of Pennsylvania and was modified to encode a C-terminal GFP fusion to allow direct identification of transduced cells. To generate the PD-L1 lentiviral vector, codon-optimized human PD-L1 was synthesized (IDT) and inserted into the BamHI site of the lentiviral vector pHR’SIN:CSW via InFusion cloning (Clontech).

### K562 culture and generation of CD19+ PD-L1+ K562 line

Cells were maintained in Iscove’s Modified Dulbecco’s Medium (IMDM) supplemented with 5% FBS and cultured according to ATCC guidelines. To generate the CD19+ PD-L1+ cell line, 1 × 10^5^ CD19+ K562 cells were transduced with lentiviral supernatant for 24 hours. Following transduction, PD-L1 expression was confirmed by flow cytometry. Bulk PD-L1+ cells were sorted to enrich for a uniform PD-L1+ population.

### Flow cytometry staining and antibodies used in this study

Fixable Live/Dead stains (Invitrogen) were utilized according to manufacturer’s instructions prior to surface staining. Following quenching and washing, surface staining was performed at 4 °C in RPMI media +1% FBS unless otherwise indicated. Antibody clones (manufacturer) utilized in this study: anti-CD8: RPA-T8 (BD), anti-CD107a: H4A3 (BD), anti-PD-1: EH12.2H7 (eBioscience), anti-PD-L1: MIH1 (BD), anti-CD69: FN50 (BD), anti-β_2_M: TU99 (BD), anti-CD19: HIB19 (BD). Data were collected on an LSR II (BD) and analyzed using FlowJo.

### CD107a staining and degranulation assay

Rested CD8+ anti-CD19 CAR T cells were incubated with CD19+ or CD19+ PD-L1+ K562s at an effector:target (E:T) ratio of 1:2 in T cell medium containing anti-CD107a, with 1 × 10^5^ CAR+ T cells seeded per well of 96-well plate. After 5 hours of co-culture cells were stained for viability (Live/Dead) and CD8, followed by flow cytometry analysis.

### *In vitro* killing assay

CD19+ (Ag^+^) or antigen irrelevant (Ag^−^) K562s were differentially labeled with 1 μM CellTrace Violet (Invitrogen) and CellTrace Far Red, respectively. CD19+ or CD19+ PD-L1+ (Ag^+^) targets were mixed 1:1 with Ag^−^ targets and 1 × 10^5^ total K562 cells seeded per well in 96-well plates. Rested CD8+ anti-CD19 CAR T cells were added to target cells at the indicated effector:target ratio. Cells were harvested, stained for Live/Dead, and analyzed by flow cytometry after 18 hours of co-culture. Specific lysis was calculated by normalization to Ag irrelevant targets and wells containing no T cells (“control wells”) after excluding non-K562 cells from the gating scheme. Specific Lysis = [(%CD19+ cells in control wells − %CD19+ cells T cell wells)/%CD19+ cells control wells] × 100.

### Cas9 RNP mediated editing and lentiviral transduction of primary human T cells

Cas9 RNP mediated editing of T cells was carried out as described^3^ with modifications. Primary human CD4+ or CD8+ T cells were thawed, rested for 24 hours in TCM followed by stimulation with plate-bound anti-CD3 (10 µg/mL; Tonbo Biosciences, clone: UCHT1) and soluble anti-CD28 (5 µg/mL; Tonbo Biosciences, clone: CD28.2) for 48 hours in T cell medium supplemented with IL-2 (30 U/mL). Electroporation was performed using the Amaxa P3 Primary Cell kit and 4D-Nucleofecter (Lonza). The recombinant *S. pyogenes* Cas9 used in this study expresses a C-terminal HA tag and two nuclear localization signal (NLS) peptides that facilitate transport across the nuclear membrane. The protein was expressed and purified as described^37^ and obtained from Macrolab, University of California, Berkeley. Cas9 was stored in 20 mM HEPES at pH 7.5, 150 mM KCl, 10% (v/v) glycerol, 1 mM TCEP at −80 °C.

Cas9 RNPs were prepared fresh for each experiment as follows: chemically synthesized tracrRNA and crRNA (Dharmacon) targeting *Pdcd1* exon 1 (PD-1 crRNA targeting sequence: 5′-CGACTGGCCAGGGCGCCTGT- 3′^3^) were re-suspended with 10 mM Tris-HCl pH 7.4 to generate 80 µM RNA stocks. crRNA and tracrRNA were mixed 1:1 and incubated 30 minutes at 37 °C to generate 40 µM crRNA:tracrRNA duplexes. An equal volume of 40 µM *S. pyogenes* Cas9-NLS was slowly added to the crRNA:tracrRNA and incubated for 15 minutes at 37 °C to generate 20 µM Cas9 RNPs. ~3 × 10^5^ stimulated T cells were re-suspended in 20 µl P3 buffer and 3 µl of 20 µM Cas9 RNP mix added. Cells were nucleofected using program EH-115. 80 µl pre-warmed TCM was added to wells and cells allowed to recover 30 minutes at 37 °C. Cells were then seeded at 1 × 10^6^ cells/mL in TCM containing Dynabeads Human T Activator anti-CD3/anti-CD28 (Invitrogen) at a bead:cell ratio of 1:1. Two-to-four hours later lentiviral supernatant was added to wells for 24–36 hours. Beads were removed 4–6 days following stimulation.

### Flow cytometry based quantification of PD-1 disruption

PD-1 surface expression was quantified using flow cytomtery 48–72 hours post nucleofection. After gating on CAR+ cells, the % reduction in PD-1+ cells was calculated as follows: 100 * [(%PD-1+ control nucleofection − %PD-1+ edited cells)/%PD-1+ control nucleofection].

For restimulation experiments, rested CD8+ CAR T cells were re-stimulated 1:1 with CD19+ K562 cells for 48 hours prior to staining for CD69 and PD-1, followed by flow cytometry analysis.

### Deep sequencing to quantify Pdcd1 disruption

The genomic regions flanking the Cas9 target site for the PD-1 target region were amplified by PCR with the following primers: 5′-TCGTCGGCAGCGTCAGATGTGTATAAGAGACAG(N/NN/NNN)CCCACCTAC CTAAGAACCATCC-3′ and 5′-GTCTCGTGGGC TCGGAGATGTGTATAAGAGACAG(N/NN/NNN)CACCCTCCCTTCAACCTGACC-3′; 150 ng genomic DNA from the edited and control samples were PCR-amplified using Kapa Hot start high-fidelity polymerase (Kapa Biosystems) according to the manufacturer’s protocol. The thermocycler setting consisted of one cycle of 98 °C for 30 s, 14 cycles of 98 °C for 20 s, 65 °C for 20 s and 72 °C for 30 s, whereby the annealing temperature was decreased by 0.5 °C per cycle, followed by 20 cycles of 98 °C for 20 s, 58 °C for 20 s, and 72 °C for 30 s and one cycle of 72 °C for 5 min. The PCR product was purified using Qiagen PCR purification kit. Nextera Index primers (Illumina) were added in a second PCR step using Kapa Hot start high-fidelity polymerase (Kapa Biosystems) with the following thermocycler program: one cycle of 98 °C for 30 s, 10 cycles of 98 °C for 20 s, 65 °C for 15 s, 72 °C for 30 s, and one cycle of 72 °C for 5 min. The resulting amplicon was purified using Qiagen PCR purification kit. Barcoded and purified DNA samples were quantified by Qubit 2.0 Fluorometer (Life Technologies), size-analyzed by BioAnalyzer (Agilent) and pooled in an equimolar ratio. Libraries were sequenced with the Illumina MiSeq desktop sequencer.

For computational analysis, reads were first trimmed using a Q15 threshold and a sliding window trimming approach. Trimmed reads were aligned to hg19 using the Burrows-Wheeler Aligner, and reads spanning a 100 bp window flanking the cut site were extracted. Quantification of the extracted reads was done using samtools mpileup of the 100 bp window flanking the target site. Insertions/deletions (INDELs) were quantified as a percentage of total trimmed and filtered reads.

### Subcutaneous tumor model in immunodeficient mice

Female NOD-*scid*-IL-2Rγ^−/−^ (NSG) mice were purchased from Jackson Laboratory and used at 6–10 weeks of age. Mice were injected subcutaneously with 5 × 10^6^ CD19+ or CD19+ PD-L1+ K562 cells in logarithmic growth phase. When tumors reached 100–1000 mm^3^ in volume, 2–7.2 × 10^6^ CAR+ CD4+ and 2–4.1 × 10^6^ CAR+ CD8+ control or PD-1 edited CAR T cells were injected intravenously as indicated in various figures. Tumor burden was monitored longitudinally using an electronic caliper.

### Statistics

Statistical tests indicated were performed using Prism software (GraphPad).

## Electronic supplementary material

Supplementary Information
