# Supplementary material for: CRISPR/Cas9-mediated PD-1 disruption enhances anti-tumor efficacy of human chimeric antigen receptor T cells
Source: Sci Rep. 2017 Apr 7;7:737. doi: 10.1038/s41598-017-00462-8 (PMC5428439; doi:10.1038/s41598-017-00462-8)
Supplement: Supplementary file 1 — Supplementary Information [file 41598_2017_462_MOESM1_ESM.pdf]

Supplementary Materials:

CRISPR/Cas9-mediated PD-1 disruption enhances anti-tumor efficacy of human chimeric antigen receptor T cells

Authors: Levi J. Rupp[1,2,3,4,†], Kathrin Schumann[3,5,6†], Kole T. Roybal[1,2,3], Rachel E. Gate[7,8], Chun J. Ye[7], Wendell A. Lim[1,2,3,4,*], Alexander Marson[3,5,6,9,10]

Affiliations:

[1]Department of Cellular & Molecular Pharmacology

[2]Center for Systems and Synthetic Biology

[3]Helen Diller Family Comprehensive Cancer Center, University of California San Francisco, San Francisco, CA 94158.

[4]Howard Hughes Medical Institute.

[5]Diabetes Center, University of California San Francisco, San Francisco, CA 94143.

[6]Department of Microbiology and Immunology, University of California San Francisco, San Francisco, CA 94143.

[7]Department of Epidemiology and Biostatistics, Department of Bioengineering and Therapeutic Sciences, Institute for Human Genetics, University of California, San Francisco, CA 94143

[8]Biological and Medical Informatics Graduate Program, University of California, San Francisco, CA 94158

[9]Division of Infectious Diseases and Rheumatology, Department of Medicine, University of California San Francisco, San Francisco, CA 94143.

[10]Innovative Genomics Initiative, University of California Berkeley, Berkeley, CA 94720.

[†]Denotes equal contribution

*Correspondence: Wendell.lim@ucsf.edu

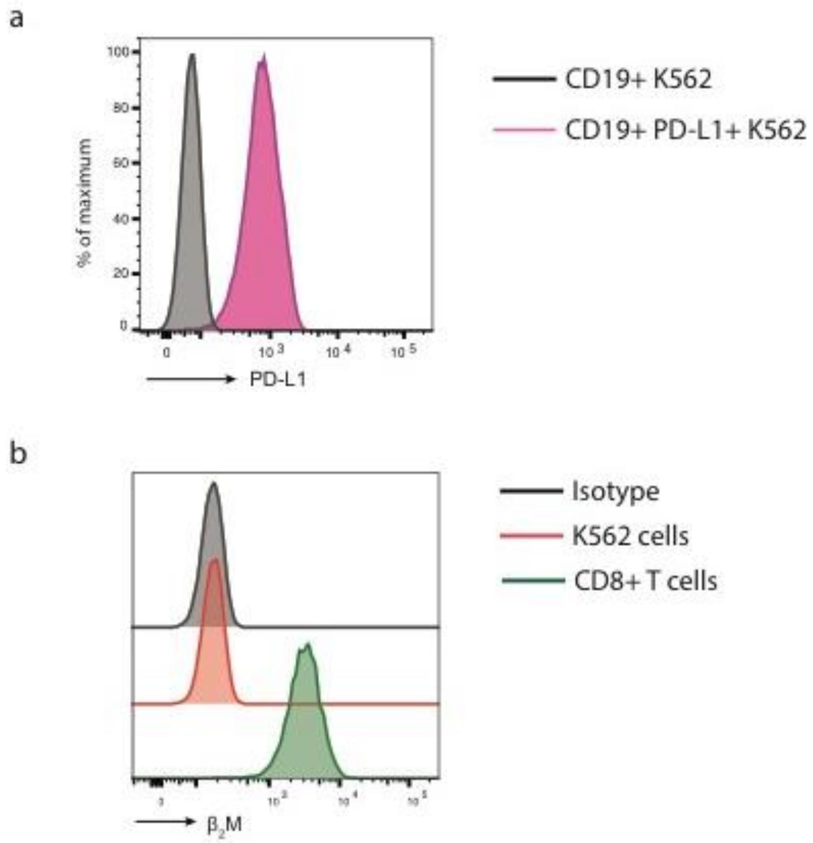

Supplementary Fig. 1. **Characterization of CD19+ PD-L1+ K562 cell line.** (**a**) Surface expression of human PD-L1 on transduced or parental CD19+ K562 cell lines. (**b**) Surface expression of beta-2-microglobulin (β_2M) in CD19+ K562 cells and control CD8+ T cells as assayed by flow cytometry.

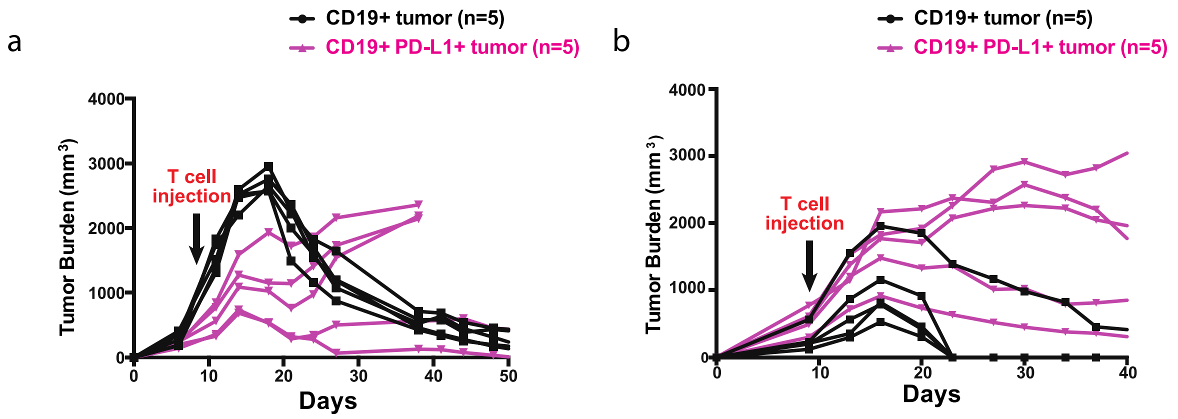

Initial Tumor Burden:	600-1000 mm^3	100-700 mm^3
T cells injected:	7.2 x 10^6 CD4+ CAR+<br>4.1 x 10^6 CD8+ CAR+	3 x 10^6 CD4+ CAR+<br>3 x 10^6 CD8+ CAR+

Supplementary Fig. 2. Impaired clearance of CD19+ PD-L1+ tumor xenografts across multiple initial tumor burdens and anti-CD19 CAR T cell doses. (**a**) NOD-*scid*-IL-2R$\gamma^{-/-}$ (NSG) mice were injected with 5 x 10^6 CD19+ or CD19+ PD-L1+ K562 cells subcutaneously. Tumors were established at 600-1000 mm^3 and mice were treated with 7.2 x 10^6 CD4+ and 4.1 x 10^6 CD8+ anti-CD19 CAR+ T cells. Tumor burden was measured longitudinally by caliper; curves show individual mice (n=5 per group). (**b**) NOD-*scid*-IL-2R$\gamma^{-/-}$ (NSG) mice were injected with 5 x 10^6 CD19+ or CD19+ PD-L1+ K562 cells subcutaneously. Tumors were established at 100-700 mm^3 and mice were treated with 3 x 10^6 CD4+ and 3 x 10^6 CD8+ anti-CD19 CAR+ T cells. Tumor burden was measured longitudinally by caliper; curves show individual mice (n=5 per group).

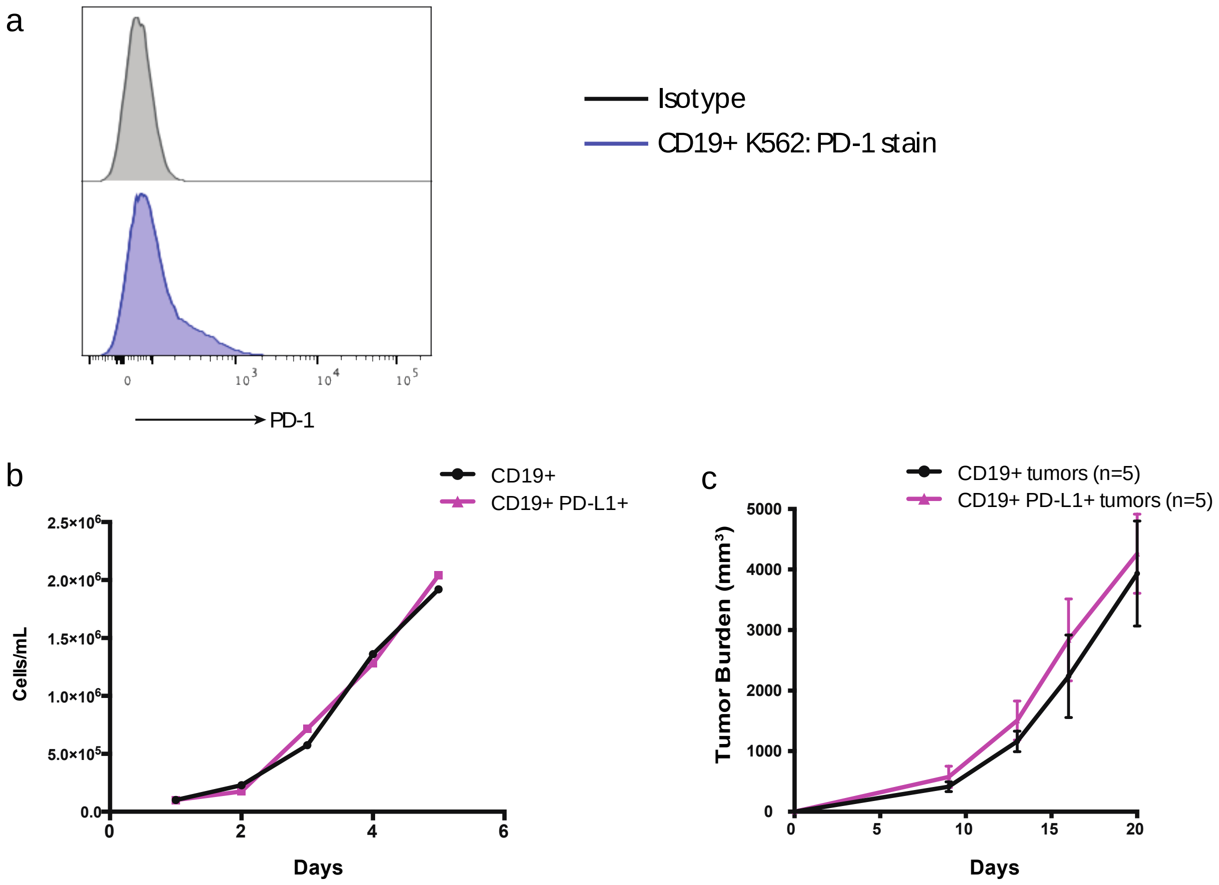

Supplementary Fig. 3. PD-1 expression and growth kinetics of CD19+ and CD19+ PD-L1+ cells lines. (**a**) Surface PD-1 expression in CD19+ K562 cultures as assayed by flow cytometry. (**b**) *In vitro* growth kinetics of CD19+ and CD19+ PD-L1+ cells. 1 x 10^5 cells/mL were seeded on day 0 and cells were counted daily for 5 days. (**c**) *In vivo* growth kinetics of CD19+ and CD19+ PD-L1+ cells. NSG mice were injected subcutaneously with 5 x 10^6 CD19+ or CD19+ PD-L1+ K562 cells and tumor burden measured longitudinally by caliper. Tumor burdens are mean ±S.D.

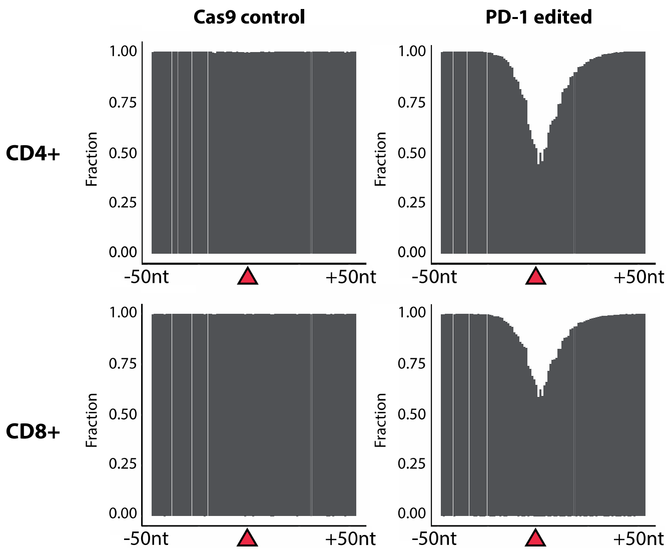

Supplementary Fig. 4. Deep sequencing of *Pdcd1* locus in control and PD-1 edited cells. Deep sequencing of the PD-1 target site in Cas9 control or PD-1 edited cells. Fraction represents the proportion of reads mapping to the reference sequence for a given nucleotide in a 100 base window spanning the cut site (denoted by ▲).

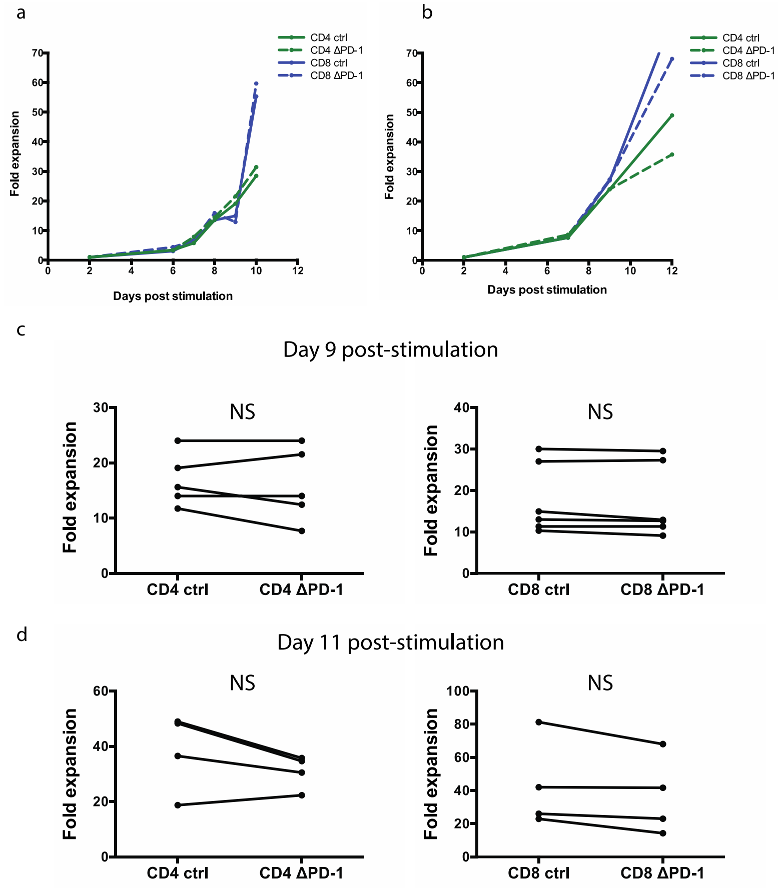

Supplementary Fig. 5. No impairment of CAR T cell expansion following *Pdcd1* editing via CRISPR. (**a**) Fold expansion of Cas9 control and PD-1 edited CD4+ or CD8+ T cells during representative experiment with no difference in expansion between treatment conditions. (**b**) Fold expansion of Cas9 control and PD-1 edited CD4+ or CD8+ T cells during representative experiment with reduced expansion of PD-1 edited cells following stimulation. (**c**) Fold expansion of Cas9 control and PD-1 edited CD4+ or CD8+ T cells 9 days after stimulation across multiple experiments. NS = not statistically significant; paired t-test. (**d**) Fold expansion of Cas9 control and PD-1 edited CD4+ or CD8+ T cells 11 days after stimulation across multiple experiments. NS = not statistically significant; paired t-test.

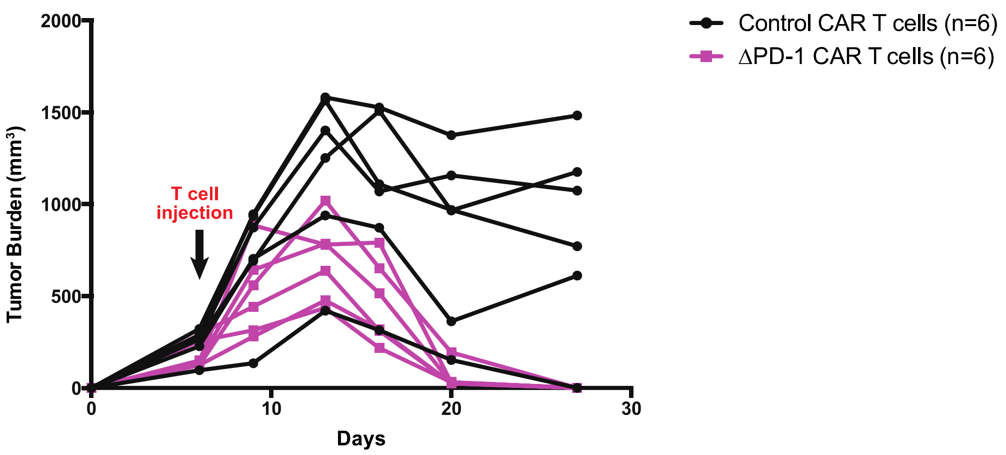

Initial Tumor Burden:	100-250 mm^3
T cells injected:	4 x 10^6 CD4+ CAR+<br>4 x 10^6 CD8+ CAR+

Supplementary Fig. 6. Improved clearance of CD19+ PD-L1+ tumor xenografts with PD-1 edited CAR T cells. Longitudinal tumor burdens for individual mice from experiment shown in Fig. 3d. NSG mice were injected with 5 x 10^6 CD19+ PD-L1+ K562 cells subcutaneously. Mice with established tumors (100-250 mm^3) were injected intravenously with 4 x 10^6 CD4+ CAR+ and 4 x 10^6 CD8+ CAR+ control T cells or PD-1 edited cells, and tumor burden measured longitudinally by caliper.

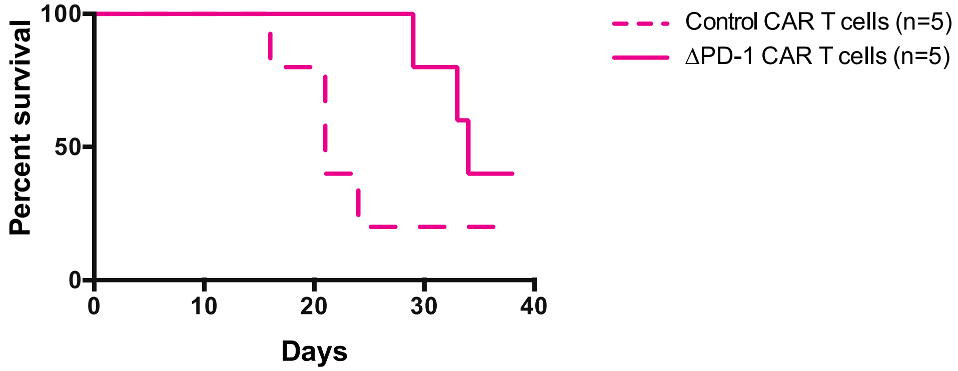

Initial Tumor Burden:	100-250 mm^3
T cells injected:	2 x 10^6 CD4+ CAR+<br>2 x 10^6 CD8+ CAR+

Supplementary Fig. 7. Kaplan-Meier curve of NSG mice with 100-250 mm^3 initial tumors treated with 2 x 10^6 CD4+ and 2 x 10^6 CD8+ control or PD-1 edited anti-CD19 CAR+ T cells. NSG mice were injected with 5 x 10^6 CD19+ PD-L1+ K562 cells subcutaneously. Mice with established tumors (100-250 mm^3) were injected intravenously with 2 x 10^6 CD4+ CAR+ and 2 x 10^6 CD8+ CAR+ control T cells or PD-1 edited cells.

a

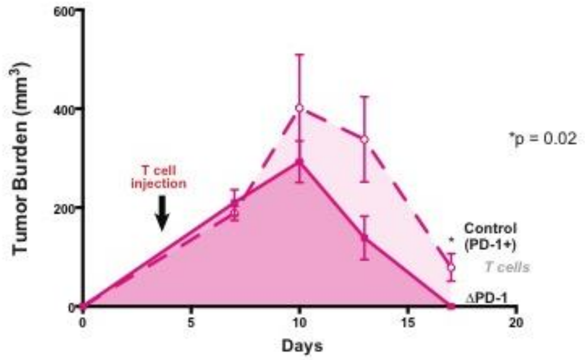

Initial Tumor Burden:	50-100 mm³
T cells injected:	5 x 10⁶ CD4+ CAR+<br>5 x 10⁶ CD8+ CAR+

b

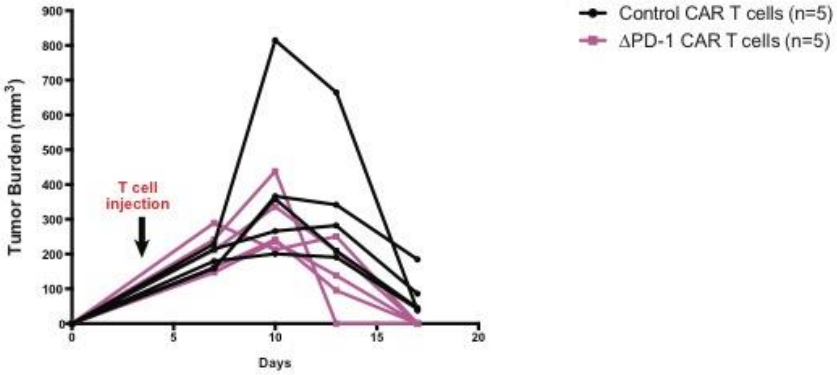

Supplementary Fig. 8. Accelerated clearance of 50-100 mm^3 tumors with PD-1 edited CAR T cells. (**a**) NSG mice were injected with 5 x 10^6 CD19+ PD-L1+ K562 cells subcutaneously. Mice with 50-100 mm^3 tumors were injected intravenously with 5 x 10^6 CD4+ CAR+ and 5 x 10^6 CD8+ CAR+ control T cells or PD-1 edited cells, and tumor burden measured longitudinally by caliper. Tumor burdens are mean ±SEM for each group (n=5 mice per group). 100% of animals receiving PD-1 edited CAR T cells cleared tumors by day 18, versus 0% in the control group (*p=0.02, Student's t-test). (**b**) Longitudinal tumor burdens for individual mice from experiment shown in (a).
